# Supplementary material for: CDCP1 knockdown suppresses PDGFRβ/AKT pathway-mediated vascular smooth muscle cell proliferation by inhibiting PDGFRβ endocytosis
Source: PeerJ. 2025 Apr 15;13:e19114. doi: 10.7717/peerj.19114 (PMC12007496; doi:10.7717/peerj.19114)
Supplement: Supplemental Information 4 [file peerj-13-19114-s004.zip › Uncropped Blots.pptx]

## Slide 1
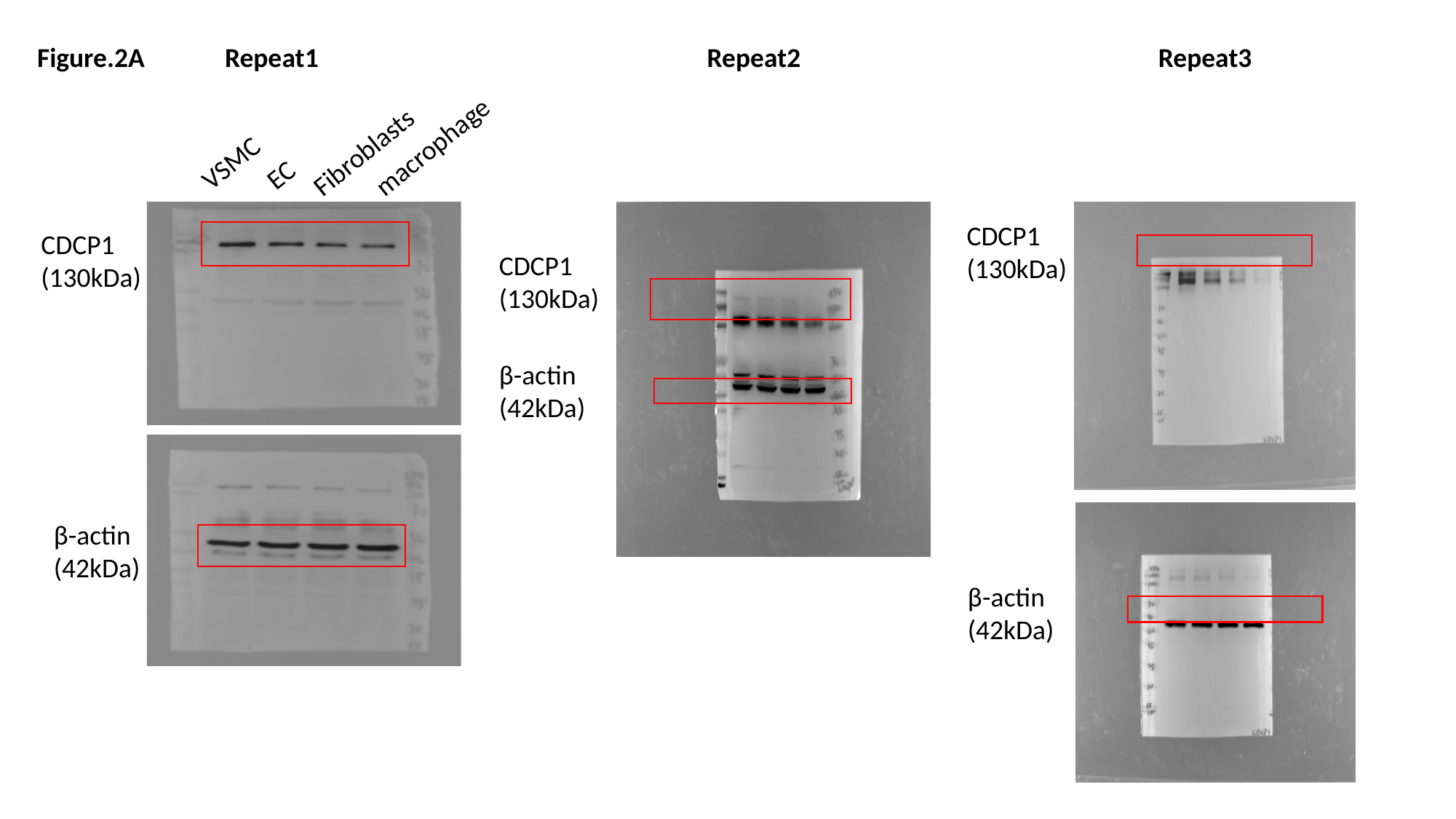

Figure.2A Repeat1 Repeat2 Repeat3
macrophage
Fibroblasts
VSMC
EC
CDCP1
(130kDa)
CDCP1
(130kDa)
CDCP1
(130kDa)
β-actin
(42kDa)
β-actin
(42kDa)
β-actin
(42kDa)

## Slide 2
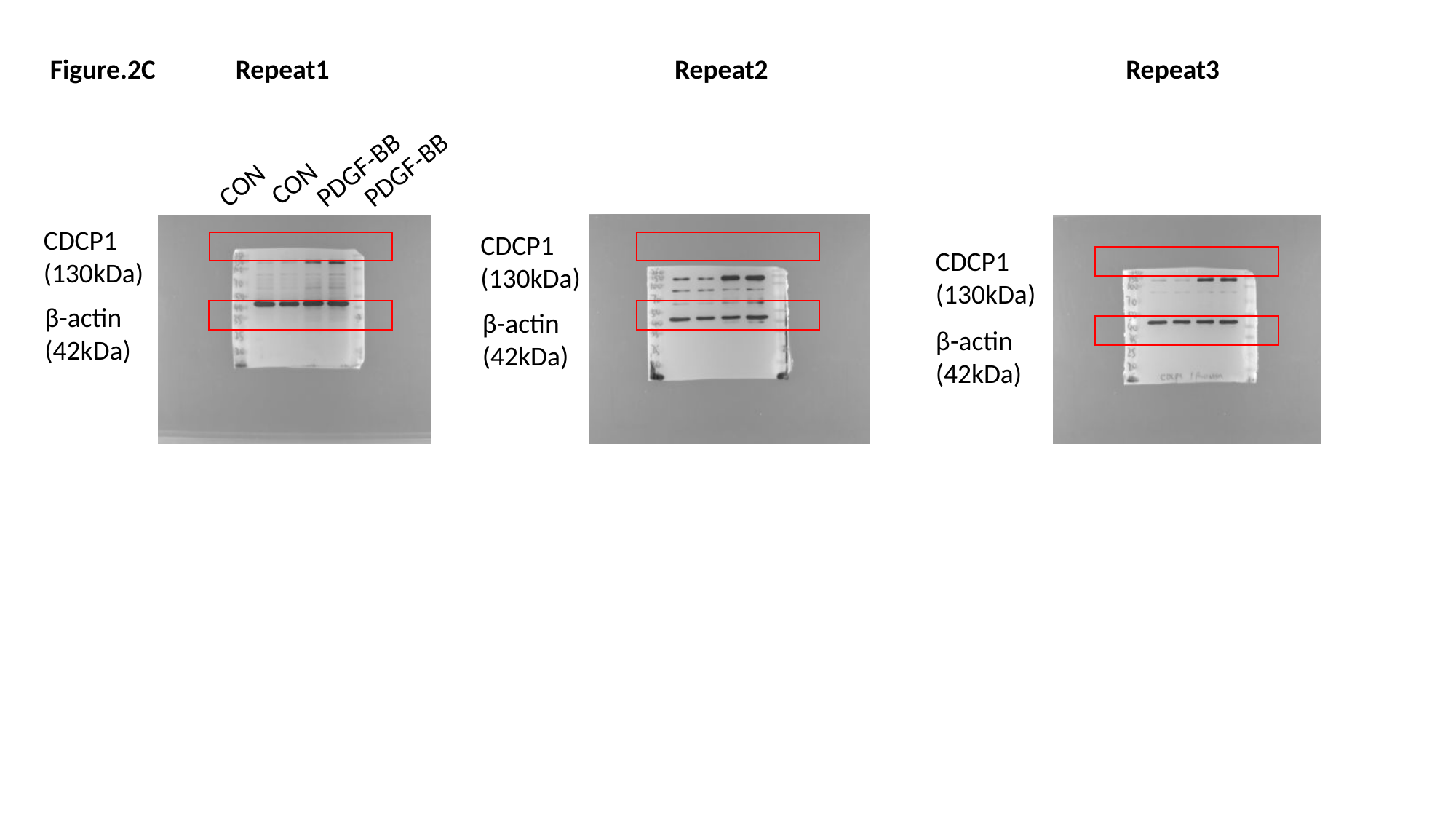

Figure.2C Repeat1 Repeat2 Repeat3
PDGF-BB
PDGF-BB
CON
CON
CDCP1
(130kDa)
CDCP1
(130kDa)
CDCP1
(130kDa)
β-actin
(42kDa)
β-actin
(42kDa)
β-actin
(42kDa)

## Slide 3
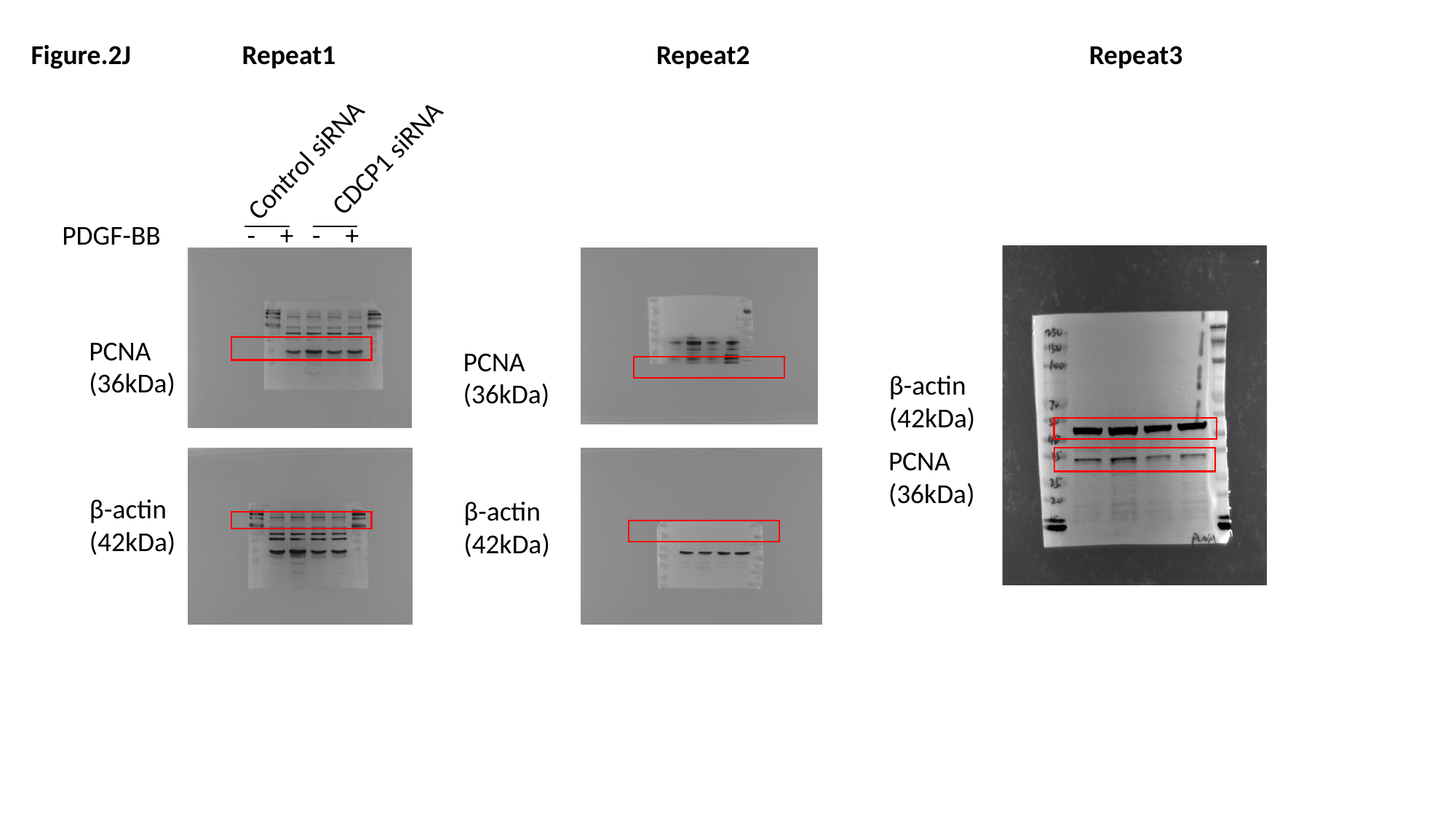

Figure.2J Repeat1 Repeat2 Repeat3
Control siRNA
CDCP1 siRNA
PDGF-BB - + - +
PCNA
(36kDa)
PCNA
(36kDa)
β-actin
(42kDa)
PCNA
(36kDa)
β-actin
(42kDa)
β-actin
(42kDa)

## Slide 4
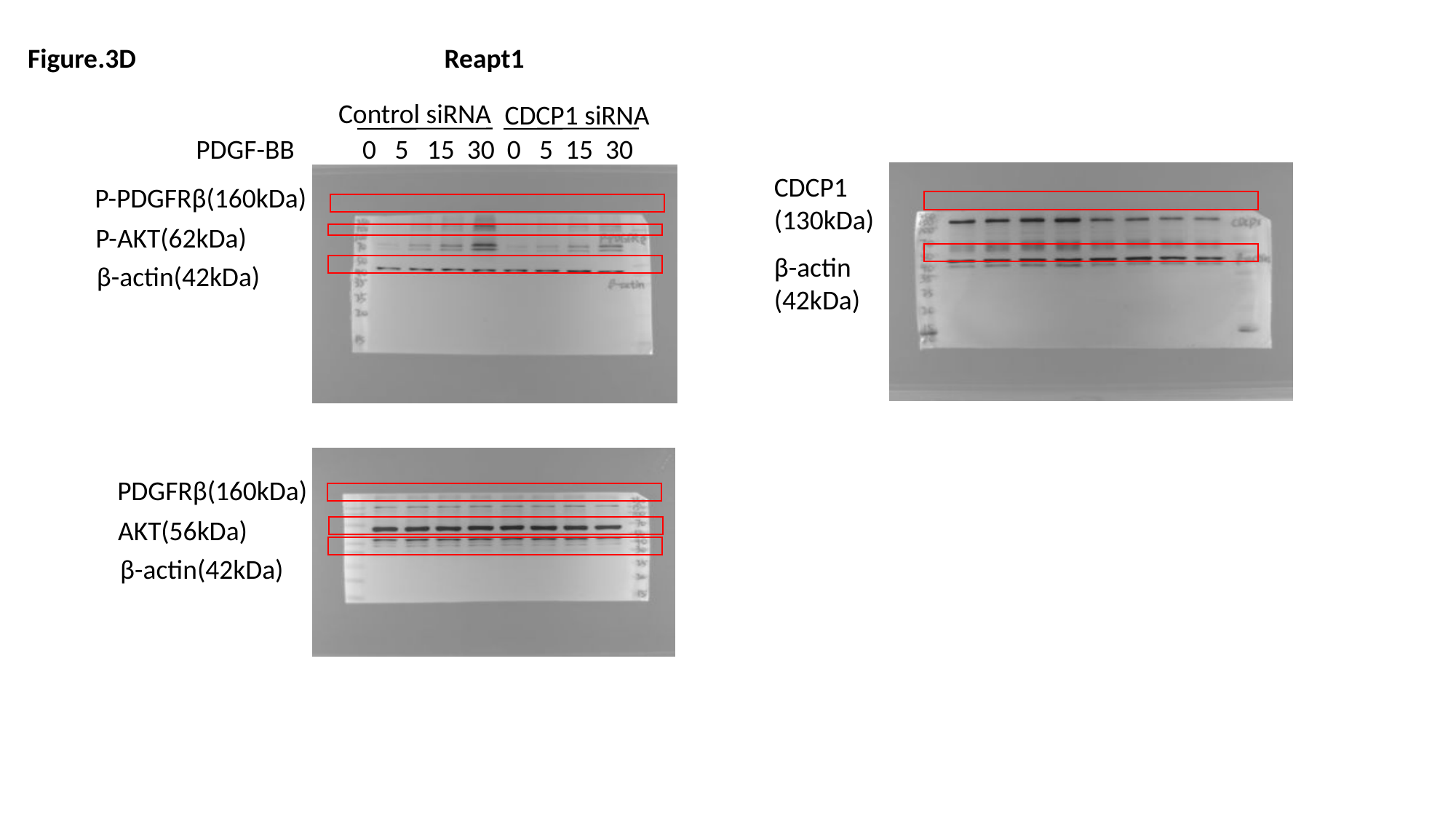

Figure.3D Reapt1
Control siRNA
CDCP1 siRNA
 PDGF-BB 0 5 15 30 0 5 15 30
CDCP1
(130kDa)
P-PDGFRβ(160kDa)
P-AKT(62kDa)
β-actin
(42kDa)
β-actin(42kDa)
PDGFRβ(160kDa)
AKT(56kDa)
β-actin(42kDa)

## Slide 5
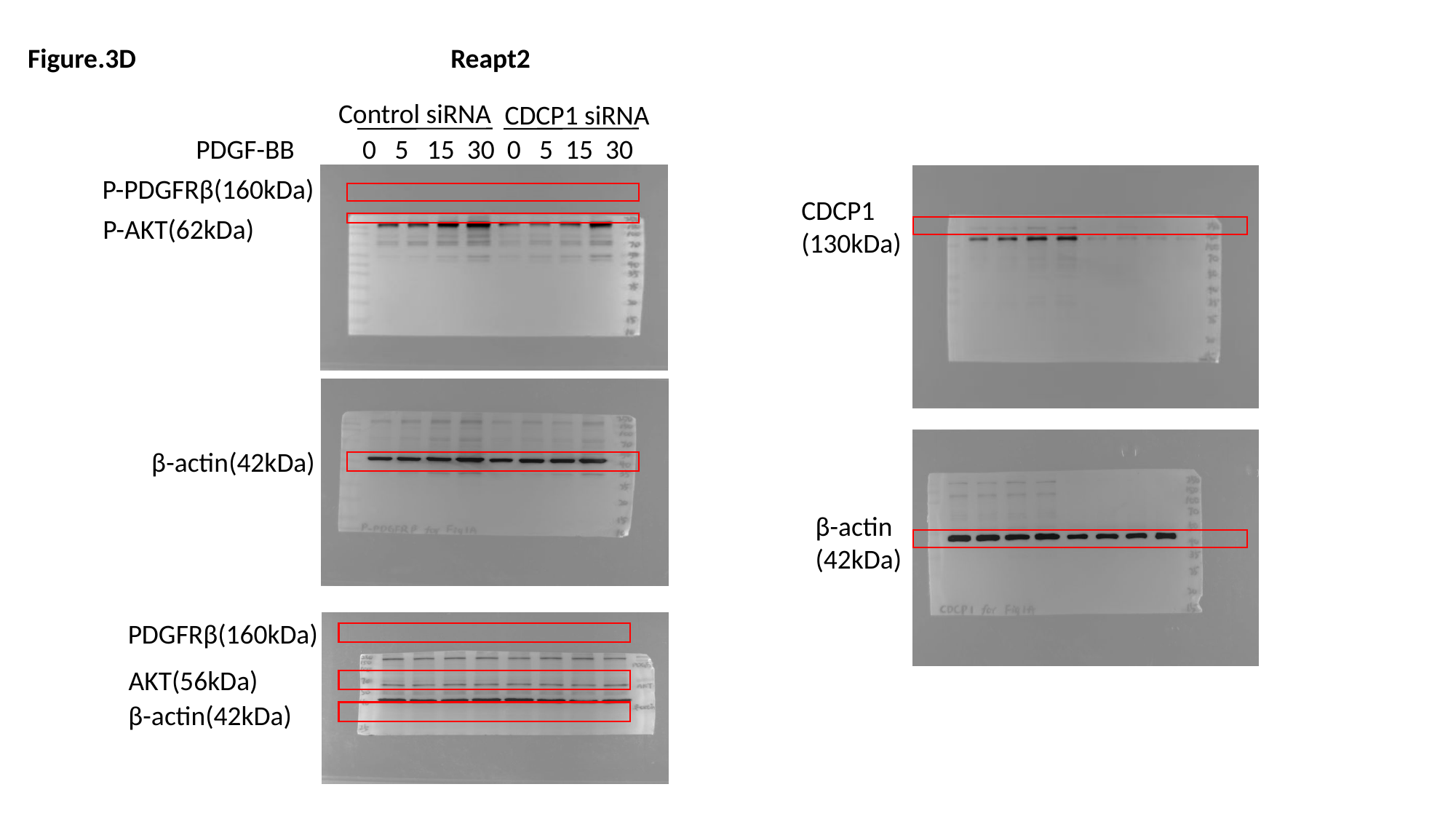

Figure.3D Reapt2
Control siRNA
CDCP1 siRNA
 PDGF-BB 0 5 15 30 0 5 15 30
P-PDGFRβ(160kDa)
CDCP1
(130kDa)
P-AKT(62kDa)
β-actin(42kDa)
β-actin
(42kDa)
PDGFRβ(160kDa)
AKT(56kDa)
β-actin(42kDa)

## Slide 6
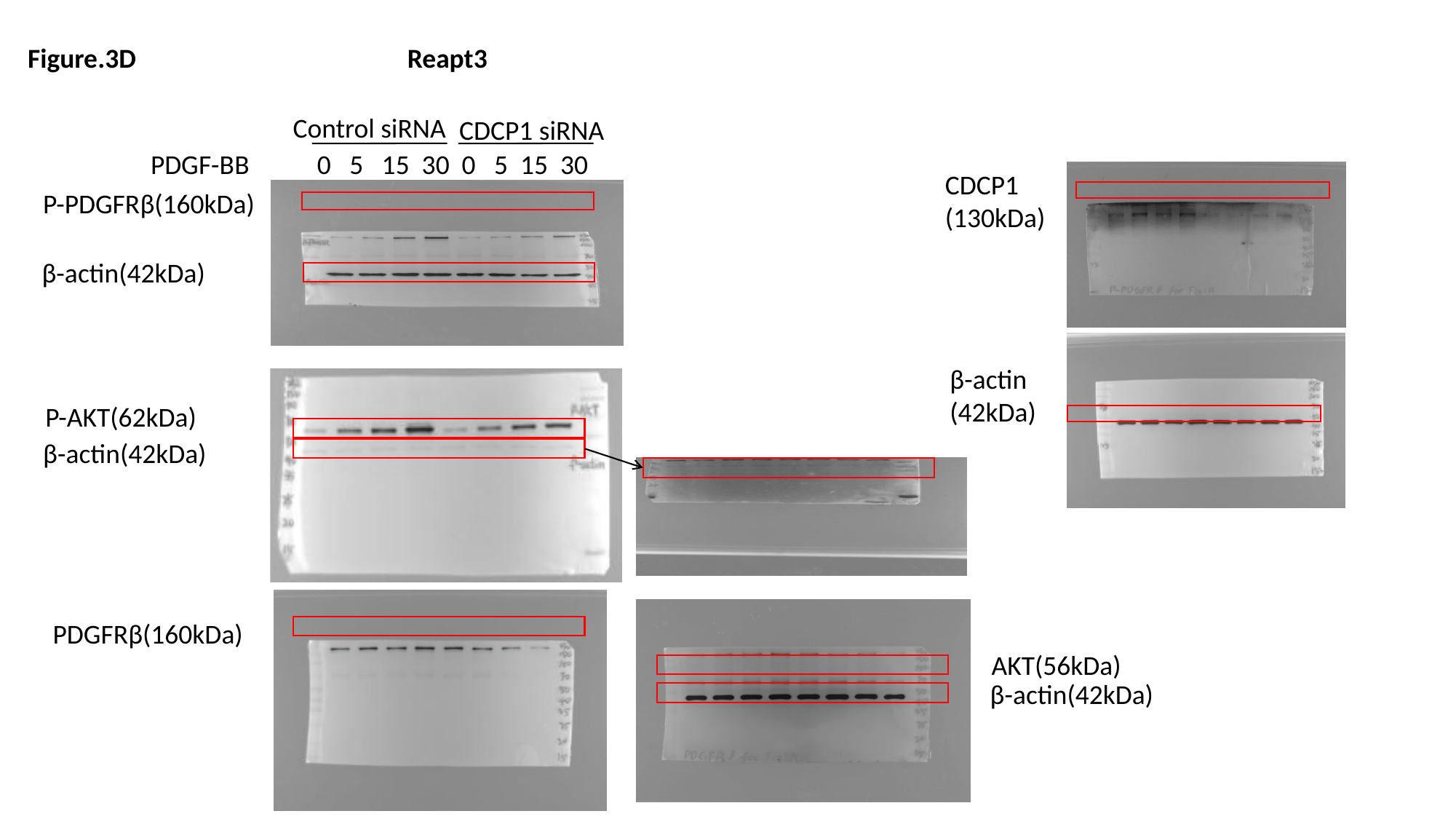

Figure.3D Reapt3
Control siRNA
CDCP1 siRNA
 PDGF-BB 0 5 15 30 0 5 15 30
CDCP1
(130kDa)
P-PDGFRβ(160kDa)
β-actin(42kDa)
β-actin
(42kDa)
P-AKT(62kDa)
β-actin(42kDa)
PDGFRβ(160kDa)
AKT(56kDa)
β-actin(42kDa)

## Slide 7
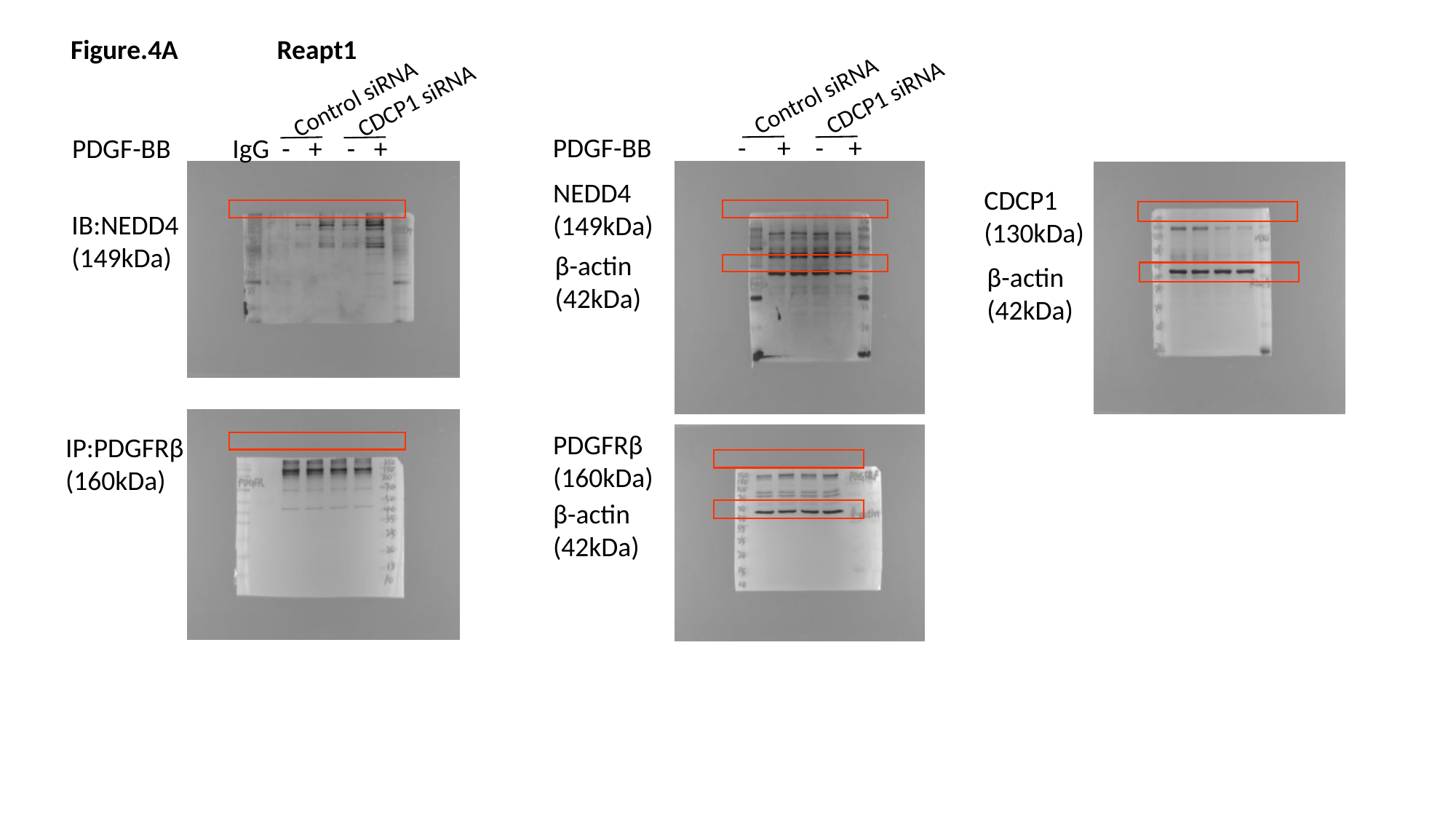

Figure.4A Reapt1
Control siRNA
CDCP1 siRNA
Control siRNA
CDCP1 siRNA
 PDGF-BB - + - +
 PDGF-BB IgG - + - +
NEDD4
(149kDa)
CDCP1
(130kDa)
IB:NEDD4
(149kDa)
β-actin
(42kDa)
β-actin
(42kDa)
PDGFRβ
(160kDa)
IP:PDGFRβ
(160kDa)
β-actin
(42kDa)

## Slide 8
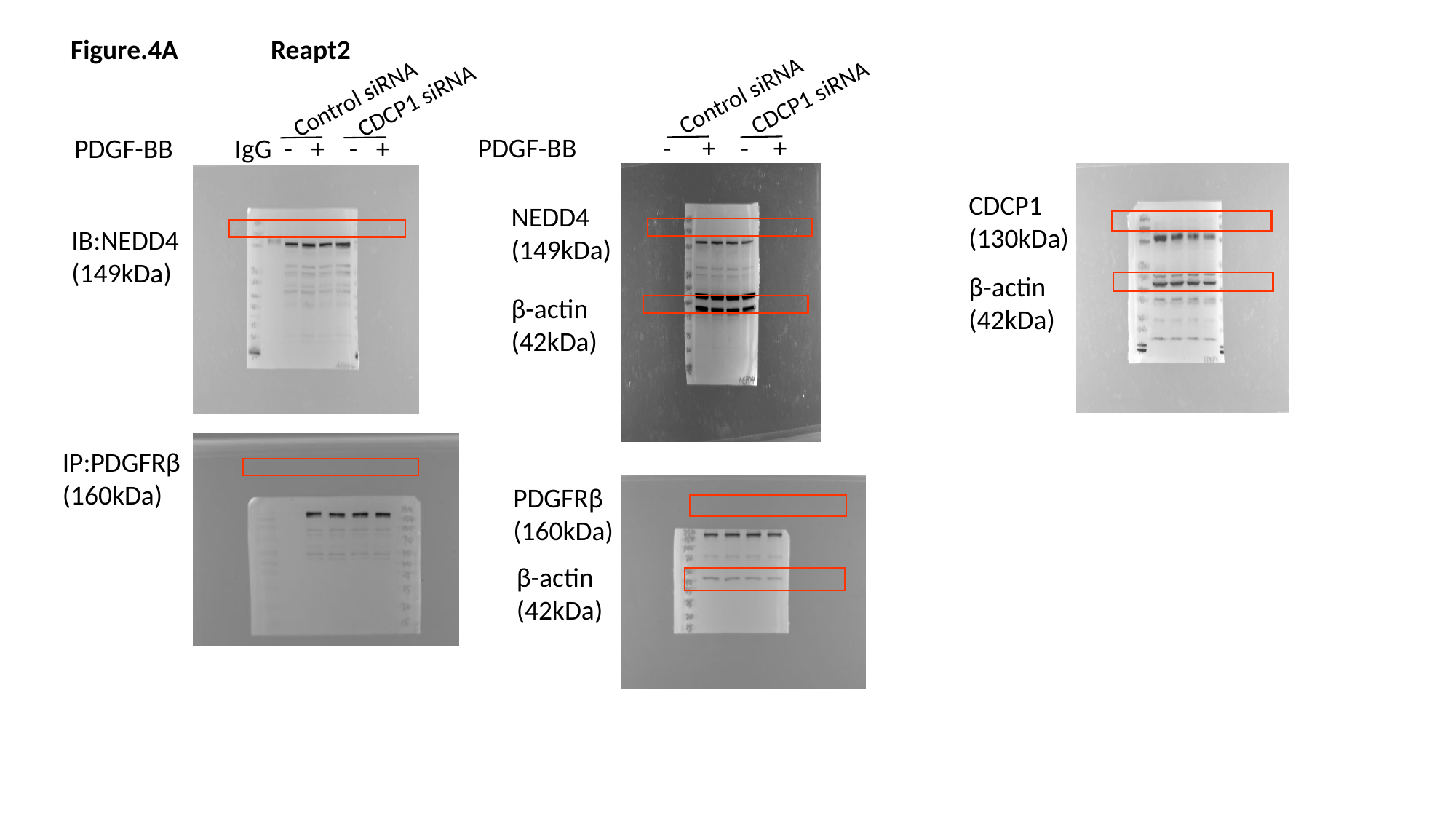

Figure.4A Reapt2
Control siRNA
CDCP1 siRNA
Control siRNA
CDCP1 siRNA
 PDGF-BB - + - +
 PDGF-BB IgG - + - +
CDCP1
(130kDa)
NEDD4
(149kDa)
IB:NEDD4
(149kDa)
β-actin
(42kDa)
β-actin
(42kDa)
IP:PDGFRβ
(160kDa)
PDGFRβ
(160kDa)
β-actin
(42kDa)

## Slide 9
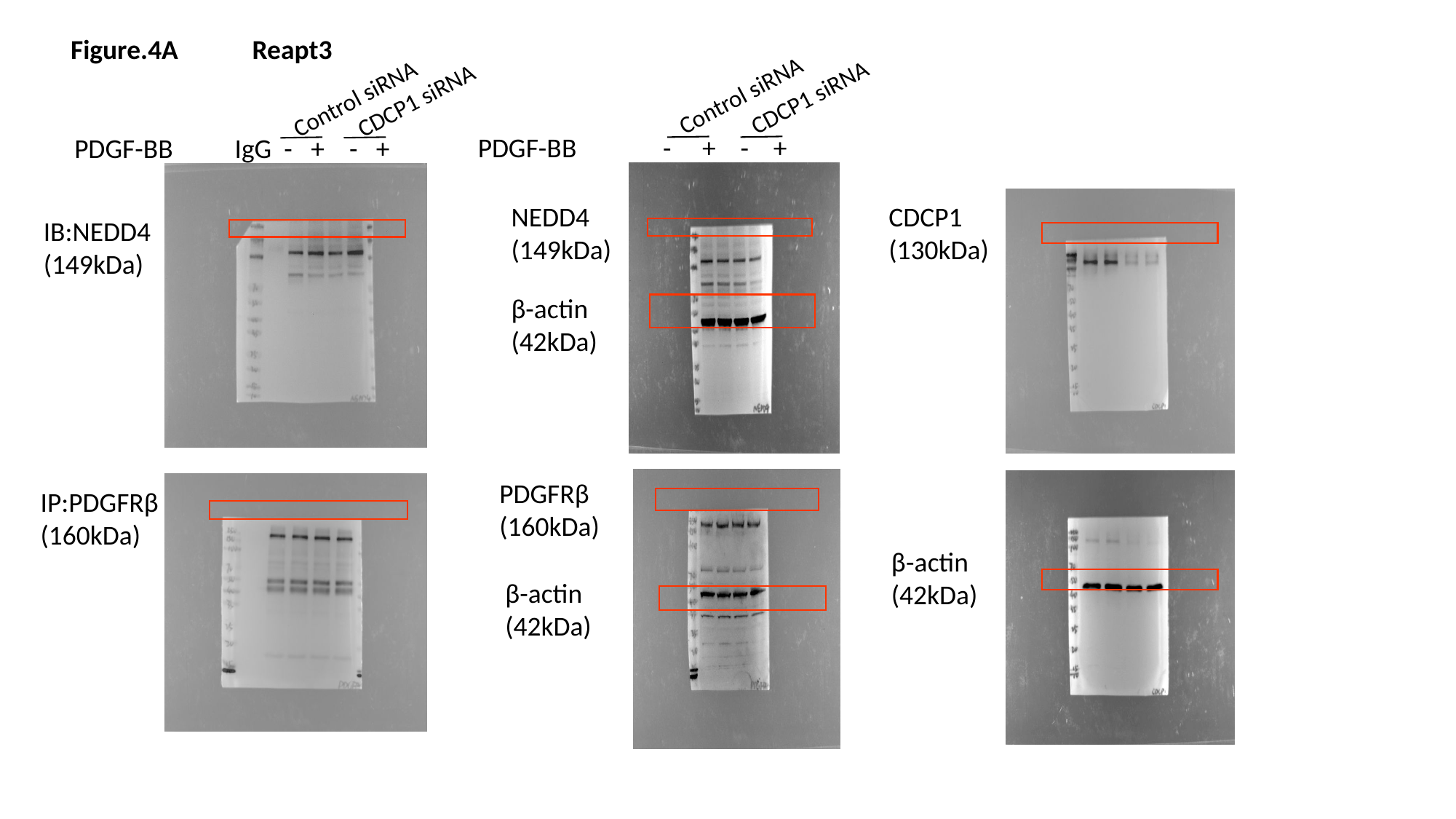

Figure.4A Reapt3
Control siRNA
CDCP1 siRNA
Control siRNA
CDCP1 siRNA
 PDGF-BB - + - +
 PDGF-BB IgG - + - +
NEDD4
(149kDa)
CDCP1
(130kDa)
IB:NEDD4
(149kDa)
β-actin
(42kDa)
PDGFRβ
(160kDa)
IP:PDGFRβ
(160kDa)
β-actin
(42kDa)
β-actin
(42kDa)

## Slide 10
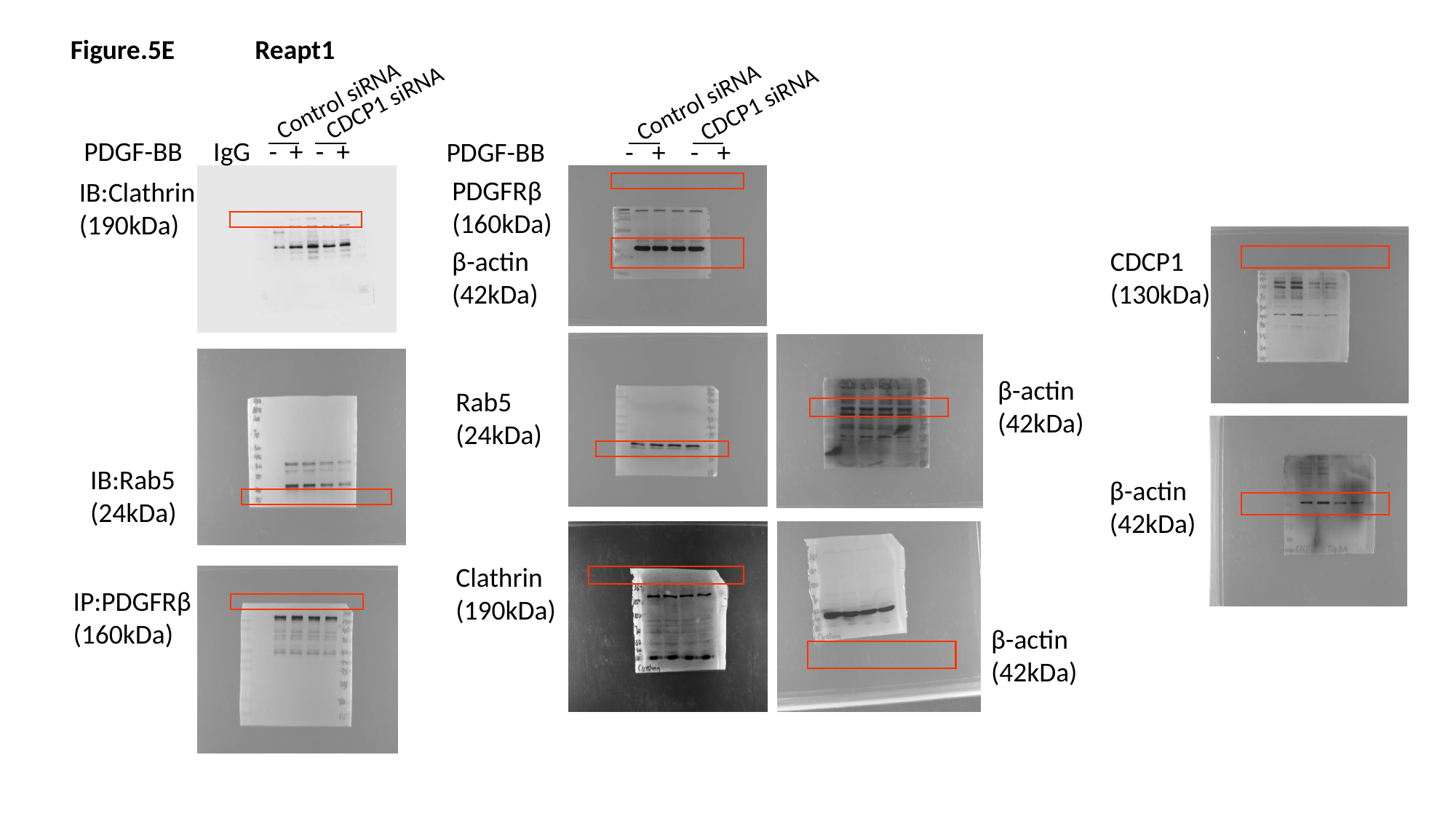

Figure.5E Reapt1
Control siRNA
CDCP1 siRNA
Control siRNA
CDCP1 siRNA
 PDGF-BB IgG - + - +
 PDGF-BB - + - +
PDGFRβ
(160kDa)
IB:Clathrin
(190kDa)
β-actin
(42kDa)
CDCP1
(130kDa)
β-actin
(42kDa)
Rab5
(24kDa)
IB:Rab5
(24kDa)
β-actin
(42kDa)
Clathrin
(190kDa)
IP:PDGFRβ
(160kDa)
β-actin
(42kDa)

## Slide 11
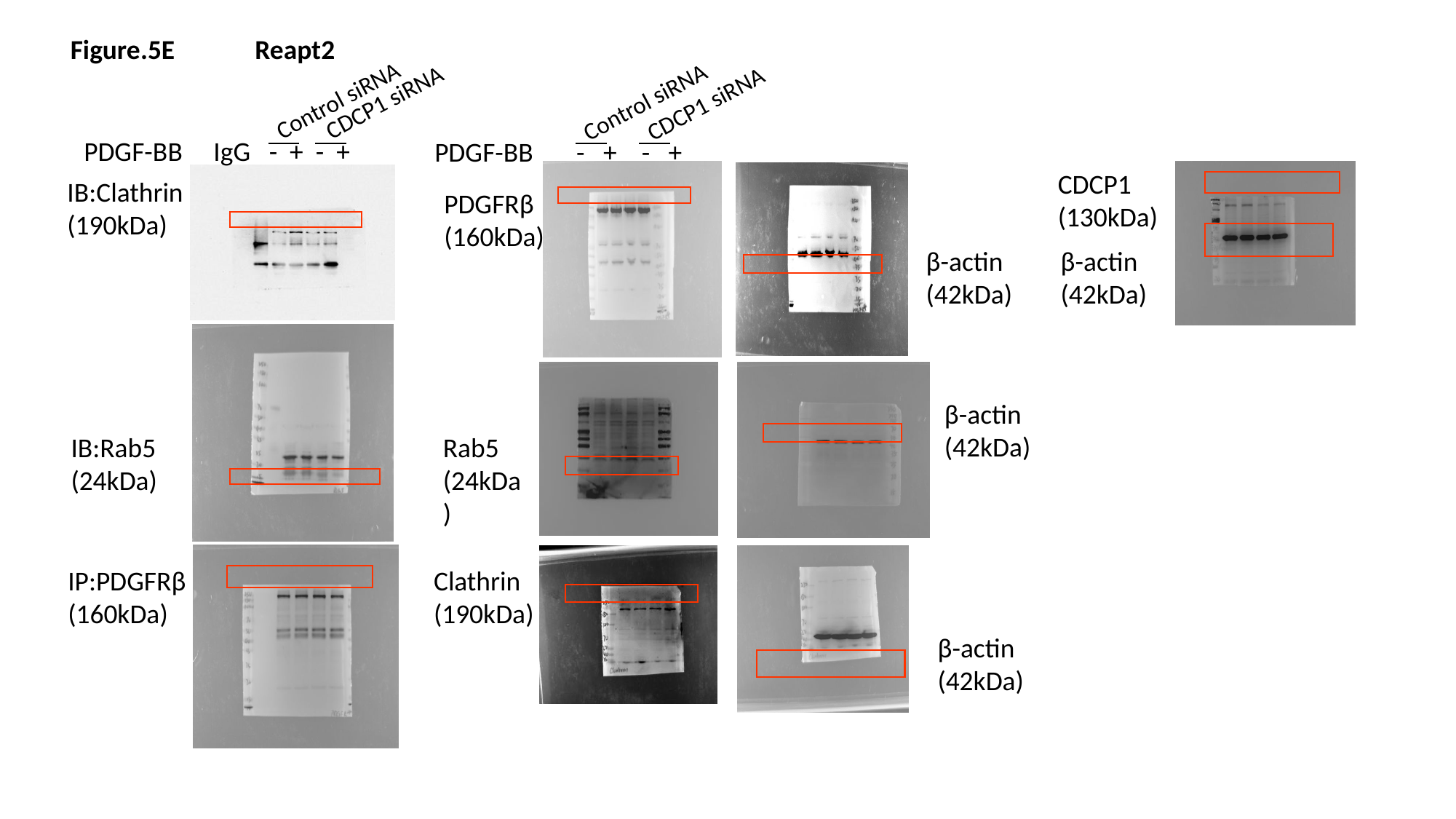

Figure.5E Reapt2
Control siRNA
CDCP1 siRNA
Control siRNA
CDCP1 siRNA
 PDGF-BB IgG - + - +
 PDGF-BB - + - +
CDCP1
(130kDa)
IB:Clathrin
(190kDa)
PDGFRβ
(160kDa)
β-actin
(42kDa)
β-actin
(42kDa)
β-actin
(42kDa)
IB:Rab5
(24kDa)
Rab5
(24kDa)
IP:PDGFRβ
(160kDa)
Clathrin
(190kDa)
β-actin
(42kDa)

## Slide 12
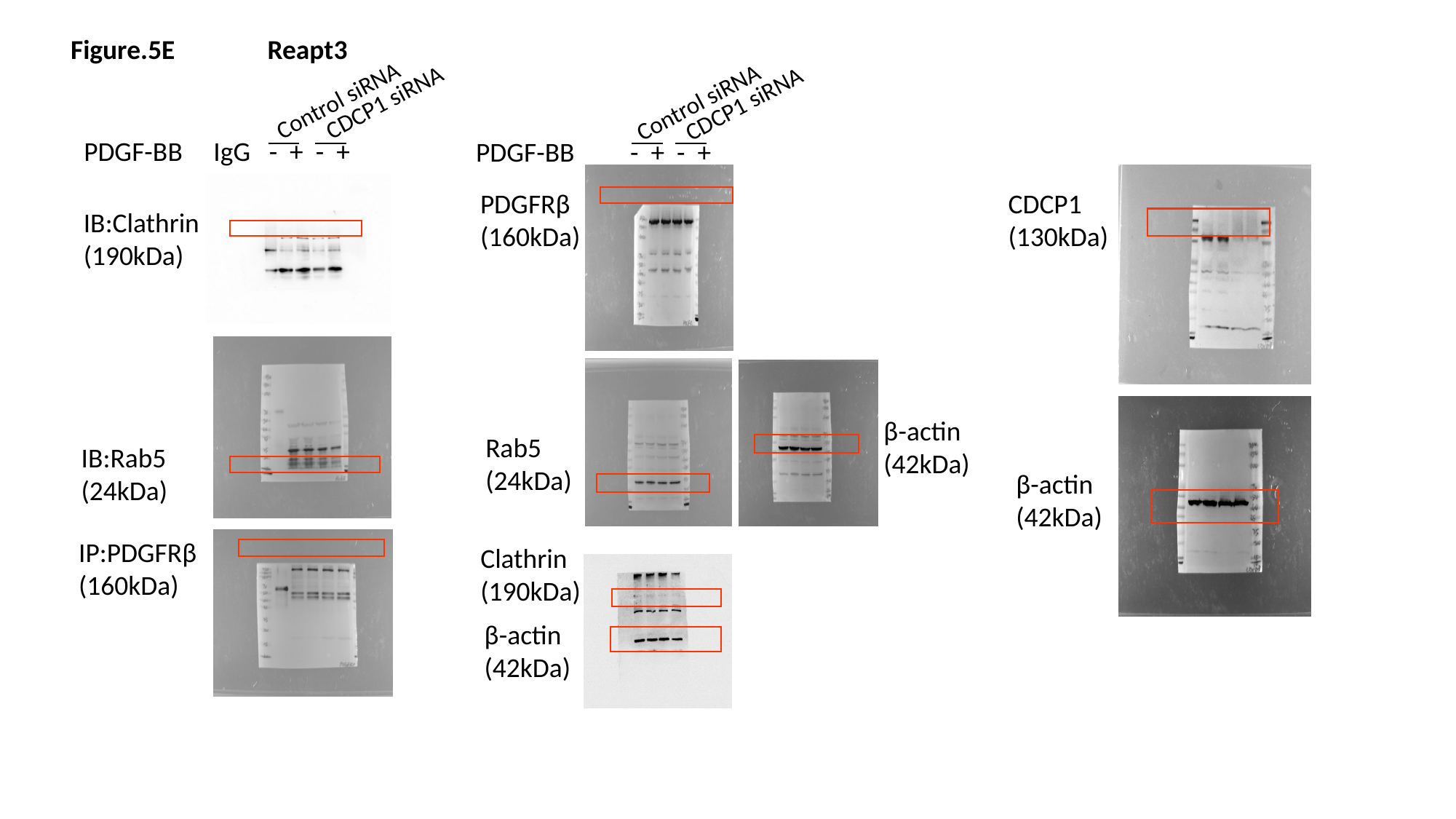

Figure.5E Reapt3
Control siRNA
CDCP1 siRNA
CDCP1 siRNA
Control siRNA
 PDGF-BB IgG - + - +
 PDGF-BB - + - +
PDGFRβ
(160kDa)
CDCP1
(130kDa)
IB:Clathrin
(190kDa)
β-actin
(42kDa)
Rab5
(24kDa)
IB:Rab5
(24kDa)
β-actin
(42kDa)
IP:PDGFRβ
(160kDa)
Clathrin
(190kDa)
β-actin
(42kDa)
