# Supplementary figures and images for: CDCP1 knockdown suppresses PDGFRβ/AKT pathway-mediated vascular smooth muscle cell proliferation by inhibiting PDGFRβ endocytosis
Source: PeerJ. 2025 Apr 15;13:e19114. doi: 10.7717/peerj.19114 (PMC12007496; doi:10.7717/peerj.19114)

A

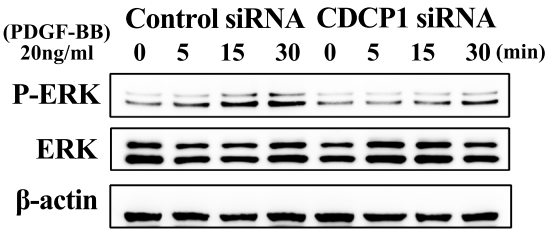

B

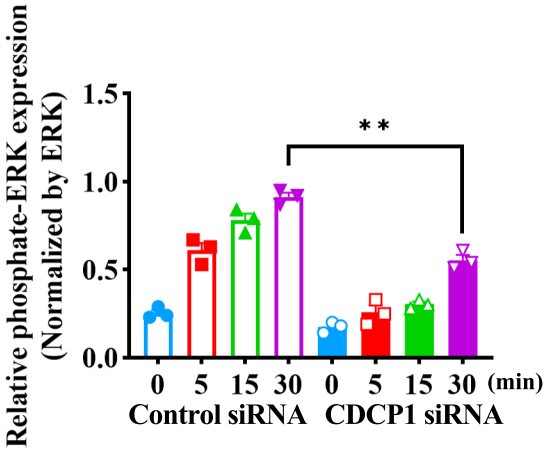

Supplement: Figure S1 [file peerj-13-19114-s005.pdf]

A

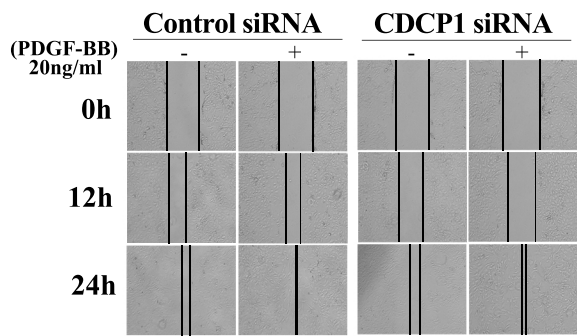

B

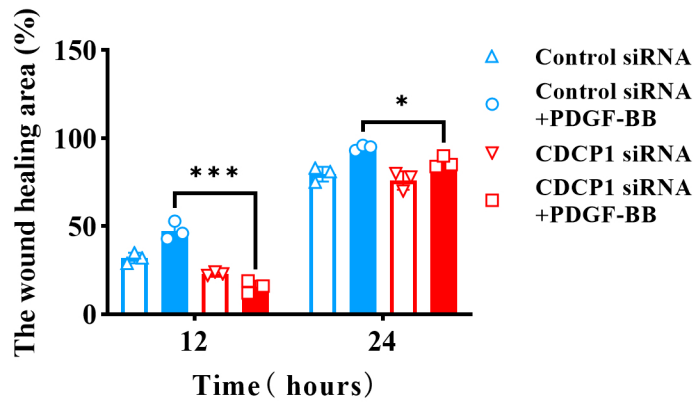

Supplement: Figure S2 [file peerj-13-19114-s006.pdf]
